# Generation of monoclonal antibodies against native viral proteins using antigen-expressing mammalian cells for mouse immunization

**DOI:** 10.1186/s12896-016-0314-5

**Published:** 2016-11-22

**Authors:** Natalie Rose, Carlos Augusto Pinho-Nascimento, Alessia Ruggieri, Paola Favuzza, Marco Tamborrini, Hanna Roth, Marcia Terezinha Baroni de Moraes, Hugues Matile, Thomas Jänisch, Gerd Pluschke, Katharina Röltgen

**Affiliations:** 1 Swiss Tropical and Public Health Institute, Molecular Immunology, Basel, Switzerland; 2 University of Basel, Basel, Switzerland; 3 Biology Institute, Fluminense Federal University, Laboratory of Molecular Virology, Niterói, Brazil; 4 Department of Infectious Diseases, University of Heidelberg, Molecular Virology, Heidelberg, Germany; 5 Oswaldo Cruz Institute, Fiocruz, Laboratory of comparative and environmental Virology, Rio de Janeiro, Brazil; 6 Section Clinical Tropical Medicine, Department of Infectious Diseases, Heidelberg University Hospital, Heidelberg, Germany

**Keywords:** Dengue virus, NS1 protein, Mouse immunization, HEK cells, Transfection, Hybridoma technology, Monoclonal antibodies

## Abstract

**Background:**

Due to their rising incidence and progressive geographical spread, infections with mosquito-borne viruses, such as dengue (DENV), chikungunya and zika virus, have developed into major public health challenges. Since all of these viruses may cause similar symptoms and can occur in concurrent epidemics, tools for their differential diagnosis and epidemiological monitoring are of urgent need.

**Results:**

Here we report the application of a novel strategy to rapidly generate monoclonal antibodies (mAbs) against native viral antigens, exemplified for the DENV nonstructural glycoprotein 1 (NS1). The described system is based on the immunization of mice with transfected mammalian cells expressing the target antigens in multiple displays on their cell surface and thereby presenting them efficiently to the host immune system in their native conformation. By applying this cell-based approach to the DENV NS1 protein of serotypes 1 (D1NS1) and 4 (D4NS1), we were able to rapidly generate panels of DENV NS1 serotype cross-reactive, as well as D1NS1- and D4NS1 serotype-specific mAbs. Our data show that the generated mAbs were capable of recognizing the endogenous NS1 protein in DENV-containing biological samples.

**Conclusion:**

The use of this novel immunization strategy, allows for a fast and efficient generation of hybridoma cell lines, producing mAbs against native viral antigens. Envisaged applications of the mAbs include the development of test platforms enabling a differentiation of the DENV serotypes and high resolution immunotyping for epidemiological studies.

**Electronic supplementary material:**

The online version of this article (doi:10.1186/s12896-016-0314-5) contains supplementary material, which is available to authorized users.

## Background

The past decades have witnessed a dramatic upsurge or emergence of arboviral diseases [1], including dengue [2, 3], chikungunya [4], and zika [5–7]. Since transmission of these viruses occurs through *Aedes aegypti* - one of the most widespread mosquito species globally - the potential for major and possibly concurrent epidemics of these viruses and other as yet unknown mosquito-borne viruses that might emerge, is overwhelming, and emphasizes the pressing need to develop vaccines and antiviral therapeutics [5]. Moreover, diagnostic tools to reliably distinguish between the various viral infections that often lead to similar clinical symptoms [8], but necessitate distinct management, are urgently required. Dengue is considered the currently most important arboviral disease [3], with an estimated 390 million cases annually [9]. Infections are caused by one of at least four antigenically distinct serotypes (DENV1-4) that vary by ~25 to 40 % at the amino acid level [10]. It has been reported that secondary infection with a heterologous serotype is associated with an increased relative risk of severe disease [10, 11]. Adequate management of severe dengue cases can greatly reduce the death rate. To date, diagnosis of the infecting serotype is of epidemiological interest, and in the future could potentially be relevant for prognosis in individual patients. Moreover, the canonical DENV serotypes appear to be antigenically more diverse than previously assumed, requiring more detailed studies of the relevance of individual antigenic determinants for clinical severity, epidemic magnitude, and DENV evolution [12].

Ever since the development of the B cell hybridoma technology in 1975 [13], the application of monoclonal antibodies (mAbs) as tools in the development of vaccines, diagnostics and therapeutics, and as general research tools has augmented [14–16]. MAbs provide a number of unique properties including the ability to bind specifically and with high affinity to almost any molecular structure as well as their availability in unlimited quantities as homogeneous reagents. Prerequisite for the generation of mAbs by the B cell hybridoma technology is the immunization of animals - most commonly mice - with the specific target antigen. In the case of proteins as target structures, immunization of mice has traditionally been accomplished by using recombinantly expressed and purified proteins, an often time-consuming and tedious endeavor. Due to their clear advantage in terms of yield and cost, simple prokaryotic expression systems, particularly *Escherichia coli*, or lower eukaryotic hosts, such as yeast, have long dominated the field of recombinant protein expression [17]. However, mammalian expression systems that are capable of providing native protein folding, and natural posttranslational modifications, have been increasingly used in the past years to reliably express proteins in their native conformation [18].

In order to meet the requirements for mAbs against viral proteins for various public health and research applications, we evaluated here a fast and highly efficient strategy of mouse immunization with native viral antigens using mammalian cells that express the recombinant proteins on their surface. This system is based on the transfection of the human embryonic kidney (HEK) suspension cell line 293F with a plasmid vector encoding the protein of interest as fusion protein containing a transmembrane domain, and thereby enabling the presentation of large amounts of the chimeric membrane-bound target antigen on the HEK cell surface in a native conformation to the immune system of animals immunized with the transfected cells. The DENV genome encodes for three structural (C, prM and E) and seven nonstructural (NS1, NS2A, NS2B, NS3, NS4A, NS4B and NS5) proteins [19]. We have chosen the DENV NS1 protein as recombinant target antigen in this study, since NS1 is an important biomarker for early diagnosis of dengue infections and has also been intensively investigated as a potential target for vaccines and antibody-based antiviral therapeutics. Depending on its glycosylation status, NS1 has a molecular weight of 46–55 kDa. The protein exists in multiple oligomeric forms and occurs as intracellular membrane-associated form, on the cell surface, or as a soluble, secreted lipoparticle [20] that can be detected in the serum of dengue patients during the acute phase of the disease [21].

The specific objectives of the present study were i) to prove the suitability of the applied immunization strategy for viral proteins, ii) to generate mAbs specifically recognizing the D1NS1 and D4NS1 proteins in their native conformation, and iii) to show that these mAbs can be used to detect the endogenous protein in DENV-containing biological samples.

## Methods

### Ethics statement

Immunization of Naval Medical Research Institute (NMRI) mice was performed in strict accordance with the rules and regulations for the protection of animal rights of the Swiss Federal Food Safety and Veterinary Office. Experiments were approved by the animal welfare committee of the Canton of Basel (authorization number 2375).

### Construction and amplification of plasmids containing DENV NS1 protein-encoding sequences

An expression plasmid pcDNA3.1 (Invitrogen, San Diego, CA) was used that has been modified to supply inserted genes with the secretion signal sequence of bee venom mellitin (BVM), a FLAG-tag, a trans-membrane (TM) domain and a hexa-His tag [22] to enable the expression of specific proteins on the surface of mammalian cells transfected with the plasmid. In addition to the DENV D1NS1 and D4NS1 protein used for immunization of mice and generation of mAbs, we also expressed the D2NS1 and D3NS1 proteins for analyses of the DENV serotype-specificity of the generated mAbs. The D1NS1 - D4NS1-encoding sequences (amino acid sequences are provided in Additional file 1: Figure S1), were cloned with restriction sites NheI and NotI into the modified pcDNA3.1 plasmid. For amplification of the constructed pcDNA3.1_BVM_D1NS1_FLAG_TM_HIS, pcDNA3.1_BVM_D2NS1_FLAG_TM_HIS, pcDNA3.1_BVM_D3NS1_FLAG_TM_HIS and pcDNA3.1_BVM_D4NS1_FLAG_TM_HIS plasmids, chemically competent *E. coli* One Shot Top10 cells (Invitrogen, San Diego, CA) were transformed according to the manufacturer’s instructions and grown in LB medium containing 100 μg/ml ampicillin. DNA was extracted and purified using the NucleoBond Xtra Maxi Plus plasmid DNA purification kit (Macherey-Nagel, Düren, Germany).

### Transfection of HEK 293F cells

The HEK cell line 293F was grown in FreeStyle 293 Expression Medium (Gibco, Grand Island, NY) in 125 ml disposable polycarbonate Erlenmeyer flasks (Corning, Oneonta, NY). Cells were maintained in a humidified incubator at 37 °C in 5 % CO_2_ on a platform shaker with rotation at 150 rpm and were passaged when the concentration of viable cells reached 2 × 10^6^ cells per ml. For the transfections, 50 μg of plasmid DNA and 150 μl of Lipofectamine 2000 Reagent (Invitrogen, Carlsbad, CA) were diluted each in 2.5 ml FreeStyle medium. DNA was added to the Lipofectamine Reagent and incubated for 5 min at room temperature. The mix was then added to 45 ml of HEK cells diluted to 10^6^ cells per ml and transfected cells were cultured as described above. After 48 h, transfected cells (D1NS1-HEK - D4NS1-HEK) were harvested. Levels of NS1 expression were assessed by Western blot analysis and immunofluorescence assay (IFA). Aliquots of 6 × 10^6^ transfected HEK cells each were stored in freezing medium (50 % fetal bovine serum, 40 % Freestyle medium and 10 % dimethylsulfoxid) in order to preserve the viability of the transfected cells at −80 °C until further use.

### Generation of mAbs against the D1NS1 and D4NS1 proteins

Stored aliquots of 6 × 10^6^ transfected D1NS1-HEK or D4NS1-HEK cells were thawed, washed and re-suspended in 0.9 % sodium chloride. Immunization of NMRI mice was conducted by intravenous injections of 10^6^ transfected HEK cells per dose in two cycles of four consecutive days with a break between cycles of 1 week. Mice with high anti-DENV NS1 IgG titers as determined by IFA of mouse sera on transfected and untransfected HEK cells and by ELISA of mouse sera on recombinant DENV NS1 (recD1NS1 and recD4NS1) proteins (AbD Serotec, Puchheim, Germany) were selected. The recombinant D1NS1 (sequence strain Nauru/Western Pacific/1974) and D4NS1 (sequence strain Dominica/814669/1981) proteins purchased from AbD Serotec are purified recombinant proteins expressed in HEK293 cells and presented in their native folded state with post-translational modifications. Selected mice received a final boost of 10^6^ transfected HEK cells each on two consecutive days. Two days after the last injections, selected mice were sacrificed and their spleens were aseptically removed. Mouse spleen cells and PAI myeloma cells were fused to generate antibody-producing hybridoma cell lines as described [23].

### DENV production

DENV serotype 1 (strain Hawaii), DENV serotype 3 (strain H87), and DENV serotype 4 (strain H241) virus stocks were prepared by virus amplification in VeroE6 cells. Virus stock titers were determined by plaque assay. In brief, VeroE6 cells were infected with serial dilutions of virus supernatants. Two hours post-infection the inoculum was replaced by serum-free MEM medium (Life Technologies, Darmstadt, Germany) containing 1.5 % carboxymethyl cellulose (Sigma-Aldrich, Taufkirchen, Germany). Five days post-infection, cells were fixed by addition of formaldehyde to a final concentration of 5 %. Cells were stained with crystal violet solution (1 % crystal violet, 10 % ethanol in H_2_O) for 30 min at room temperature and rinsed extensively with H_2_O. Infectious titers were calculated considering the corresponding dilution factor. DENV serotype 2 (strain New Guinea C) virus stock was produced by electroporation of BHK-21 cells with in vitro transcripts and further amplification of the BHK-21 supernatant in VeroE6 cells as described above. Virus stock titer was determined by plaque assay and cells were fixed 7 days post infection.

### Western blot analysis with HEK cell and VeroE6 cell lysates

In order to prepare HEK cell protein lysates, aliquots of 6 × 10^6^ transfected or untransfected (negative control) HEK cells were thawed, washed and re-suspended in 600 μl of RIPA buffer containing 20 mM Tris HCl, 137 mM NaCl, 10 % glycerol, 1 % NP-40, 0.25 % sodium deoxycholate and a protease inhibitor cocktail (cOmplete Mini; Roche, Mannheim, Germany). After incubation for 30 min on ice, the mix was centrifuged at 10’000 rpm for 10 min and the supernatant was stored at −20 °C.

For VeroE6 cell lysates, 10^6^ cells were infected with a multiplicity of infection of 10 for 36 h (DENV serotypes 1, 2 and 4) and 48 h (DENV serotype 3). Cells were lysed with 100 μl ice-cold lysis buffer (50 mM Tris HCl pH 7.4, 150 mM NaCl, 1 % Triton X-100, 60 mM β-glycerophosphate, 15 mM 4-nitrophenylphosphate, 1 mM sodium orthovanadate, 1 mM sodium fluoride, and protease inhibitor cocktail).

For SDS-PAGE, 2 μg of HEK cell lysates per lane were separated on NuPAGE Novex 4–12 % Bis-Tris Gels (Invitrogen, Carlsbad, CA) with addition of NuPage Reducing Agent (Invitrogen, Carlsbad, CA) and heating of the samples for 5 min at 95 °C (reducing conditions). Lysates of DENV-infected VeroE6 cells were separated on NuPAGE Novex 4–12 % Bis-Tris Zoom Gels under non-reducing conditions (without addition of reducing agent and without heating of samples). After electrophoresis, protein bands were either visualized directly with aqua-staining or were transferred onto nitrocellulose membranes for Western blot analysis using an iBlot Gel Transfer Device (Invitrogen). Membranes were blocked with 5 % non-fat dry milk in PBS containing 0.1 % Tween 20 and cut into strips, if required. Membranes or membrane strips were then incubated with anti-hexa-His tag, anti-tubulin or anti-DENV NS1 mAbs, washed with PBS containing 0.1 % Tween 20 and thereafter incubated with horseradish peroxidase-conjugated goat anti-mouse IgG antibodies (γ-chain specific; Southern Biotech, Birmingham, AL). After a second washing step, bands were visualized by chemiluminescence using ECL Western Blotting substrate (Thermo Scientific, Rockford, IL).

### Immunofluorescence staining of HEK cells

For IFA, an aliquot of 6 × 10^6^ transfected HEK cells was thawed, washed and re-suspended in 600 μl of PBS. Each well of 12-well multi-test glass slides (MP Biomedicals, Inc., Illkirch, France) was coated with 2 μl of the cell suspension. After air-drying under a sterile hood, cells were fixed in methanol for 15 min. Dried wells were then incubated with either hybridoma cell culture supernatant or purified mAbs diluted in PBS containing 0.5 % BSA for 20 min in a wet chamber at 37 °C. Following rinsing, washing twice for 5 min in PBS containing 0.05 % Tween 20 and rinsing in distilled H_2_O, dried wells were incubated with Alexa568-conjugated goat anti-mouse IgG antibodies (KPL, Gaithersburg, MD) diluted in PBS containing 0.5 % BSA for 20 min in a wet chamber at 37 °C. After washing as described, dried slides were mounted with ProLong Gold antifade reagent with DAPI (Life technologies, Eugene, OR) and covered with a cover slip. Analyses were performed on a Leica CTR500 fluorescence microscope and images were taken with a Leica DFC345 FX digital camera (Leica Microsystems, Heerbrugg, Switzerland).

### Conventional ELISA on recombinant NS1 proteins

96-well Nunc-Immuno Maxisorp plates (Thermo Scientific, Roskilde, Denmark) were coated overnight at 4 °C in a wet chamber with 50 μl PBS containing recombinant DENV NS1 (recD1NS1 and recD4NS1) protein (AbD Serotec, Puchheim, Germany) at a concentration of 1 μg/ml or an unrelated hexa-His tagged protein (MUL_3720 [24]) serving as a control. After washing with distilled H_2_O containing 0.01 % Tween 20, wells were blocked with 5 % non-fat dry milk in PBS for 1 h at 37 °C. Subsequently, wells were incubated with mouse antisera or hybridoma cell culture supernatant diluted in PBS containing 0.5 % milk and 0.05 % Tween20 for 1 h at 37 °C. After washing as described, horseradish peroxidase-conjugated goat anti-mouse IgG antibodies (γ-chain specific; Southern Biotech, Birmingham, AL) in PBS containing 0.5 % milk and 0.05 % Tween20 were incubated for 1 h at 37 °C. Plates were washed and developed with TMB Microwell Peroxidase Substrate (KPL, Gaithersburg, MD). The reaction was stopped using 0.5 M sulfuric acid. The absorbance was measured at 450 nm in a microplate reader (Tecan Sunrise, Grödig, Austria).

### Anti-hexa-His tag capture ELISA

96-well Nunc-Immuno Maxisorp plates (Thermo Scientific, Roskilde, Denmark) were coated overnight at 4 °C in a wet chamber with 50 μl PBS containing anti-hexa-His tag antibodies at a concentration of 5 μg/ml. All following steps were carried out on the next day and at room temperature. After washing with distilled H_2_O containing 0.01 % Tween 20, wells were blocked with 5 % non-fat dry milk in PBS for 2 h. Subsequently, wells were incubated with D1NS1-HEK - D4NS1-HEK cell whole protein lysates diluted in PBS for 2 h. After another washing step, wells were incubated with biotinylated anti-NS1 mAbs at a concentration of 5 μg/ml diluted in PBS containing 0.5 % milk for 2 h. Wells were washed and incubated with horseradish peroxidase-conjugated Streptavidin in PBS containing 0.5 % milk for 1 h. Plates were washed and developed with TMB Microwell Peroxidase Substrate (KPL, Gaithersburg, MD). The reaction was stopped using 0.5 M sulfuric acid. The absorbance was measured at 450 nm in a microplate reader (Tecan Sunrise, Grödig, Austria).

### Commercial DENV NS1 capture ELISA

Lysates of VeroE6 cells infected with different DENV serotypes were analyzed for their NS1 content by a DENV NS1-specific capture ELISA (EUROIMMUN, Luzern, Switzerland) according to the manufacturer’s instructions. The three calibrator samples supplied with the kit were applied to assess relative units of the NS1 protein present in the samples.

## Results

### Expression of D1NS1 and D4NS1 on the surface of transfected HEK 293F cells

**Fig. 1:**
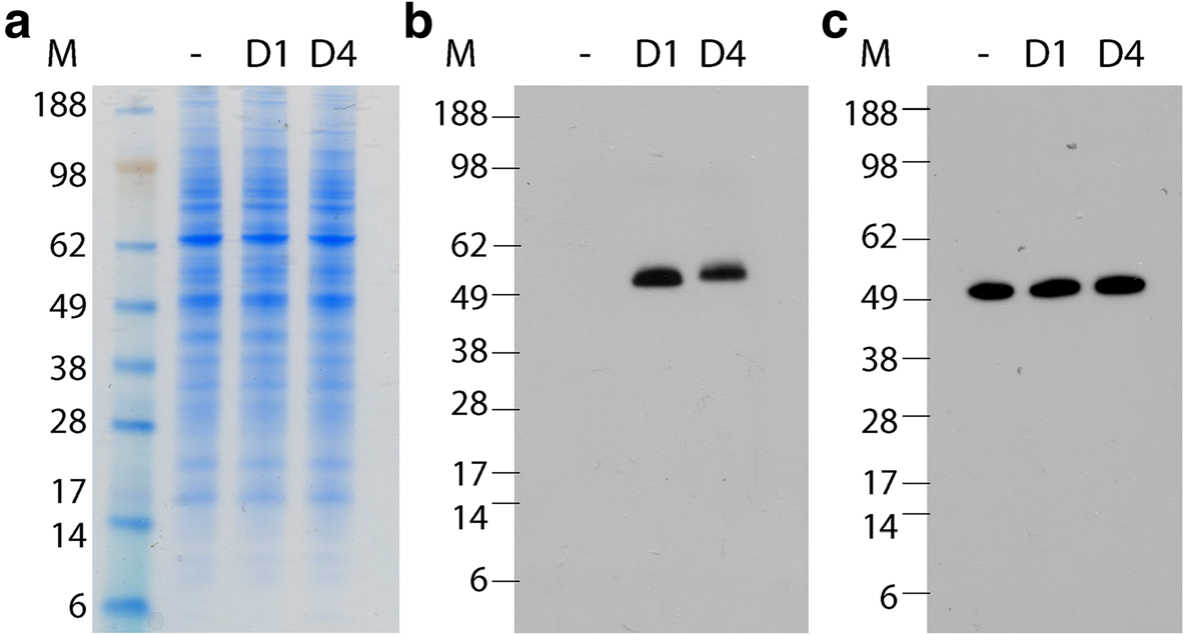
Expression of D1NS1 and D4NS1 by transfected HEK cells. HEK cell lysates were analyzed by aqua-staining and Western blot analysis after separation on NuPAGE Novex 4–12 % Bis-Tris Gels under reducing conditions (addition of reducing agent and heating of the samples). While aqua-staining of lysates prepared from HEK-derived cell lines expressing D1NS1 (D1) and D4NS1 (D4) showed no additional band of the predicted size of the NS1 proteins, as compared to the lysates of untransfected HEK cells (−) (**a**), Western blot analysis using anti-hexa-His tag antibodies confirmed the expression of D1NS1 and D4NS1 by the HEK cells (**b**). Western blotting using anti-tubulin antibodies was performed as a control for the amount of cellular proteins of the untransfected and transfected HEK cell lysates loaded on the gels (**c**). M = molecular weight marker in kDa

**Fig. 2:**
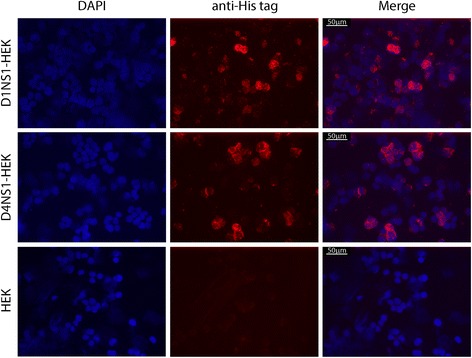
Localization of D1NS1 and D4NS1 on the surface of transfected HEK cells. IFA of methanol fixed D1NS1-HEK, D4NS1-HEK and untransfected HEK cells using mouse anti-hexa-His tag mAbs and Alexa568-labelled anti-mouse IgG antibodies. Nuclei were stained with DAPI

After transfection of HEK cells with the NS1 fusion protein-encoding plasmids pcDNA3.1_BVM_D1NS1_FLAG_TM_HIS or pcDNA3.1_BVM_D4NS1_FLAG_TM_HIS, the expression of D1NS1 and D4NS1 by the transfectants was analyzed. No elevated expression levels of a protein corresponding in size to the NS1 protein was visible upon aqua-staining of the SDS-PAGE separated total proteins of D1NS1-HEK and D4NS1-HEK cell lysates when compared to a lysate of untransfected HEK cells (Fig. 1a). However, Western blot analysis with anti-hexa-His tag antibodies (Fig. 1b) confirmed the specific expression of the NS1 fusion proteins (a band at the expected size for the monomer NS1 of 46–55 kDa [20]) by the transfectants. In combination with Western blot analysis with anti-tubulin antibodies serving as a loading control (Fig. 1c), these analyses revealed no significant difference between expression levels of D1NS1 and D4NS1. IFA of D1NS1-HEK, D4NS1-HEK and untransfected HEK cells with anti-hexa-His tag antibodies demonstrated the cell surface localization of the expressed NS1 fusion proteins with similar staining intensities for D1NS1 and D4NS1 (Fig. 2).

### Development of DENV NS1-specific antibody responses in mice by immunization with transfected HEK cells

**Fig. 3:**
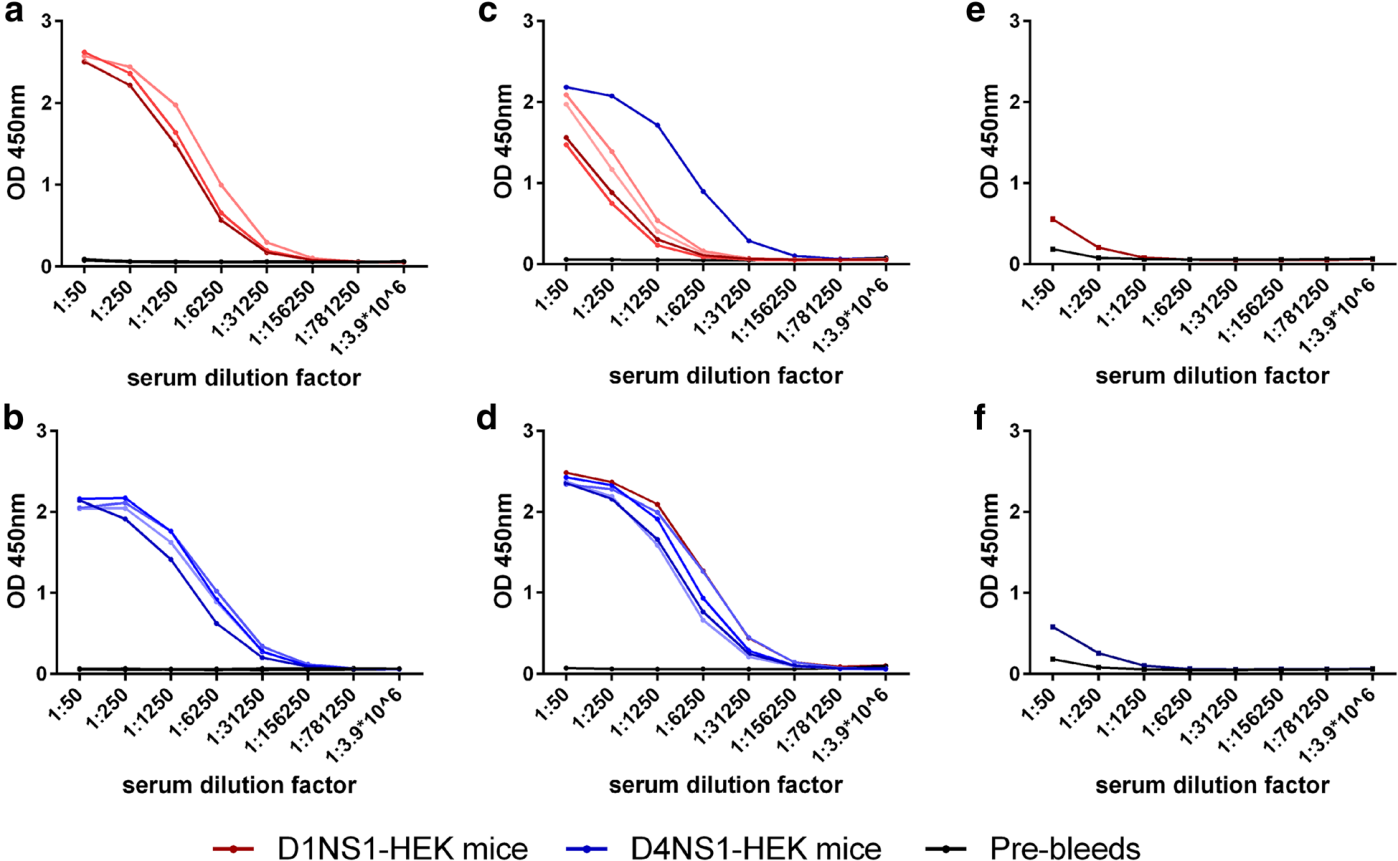
ELISAs showing humoral immune responses of mice immunized with D1NS1-HEK and D4NS1-HEK cells. **a** and **b**: Mouse antisera of D1NS1-HEK (red, panel **a**) and D4NS1-HEK (blue, panel **b**) immunized mouse groups (4 mice each) were analyzed on recombinant D1NS1 and recombinant D4NS1, respectively. **c** and **d**: Serotype cross-reactive humoral immune responses of D1NS1-HEK (red, panel **c**) and D4NS1-HEK (blue, panel **d**) mouse groups were analyzed on recombinant D4NS1 and recombinant D1NS1, respectively. Blue line (panel **c**) and red line (panel **d**) = pool of D4NS1-HEK and D1NS1-HEK mouse antisera, respectively, to enable direct comparison with the level of serotype-specific immune responses. **e** and **f**: Analysis of pooled mouse sera (red = D1NS1-HEK (**e**), blue = D4NS1-HEK (**f**)) on an unrelated hexa-His tagged protein (MUL_3720) as a control for humoral responses against the hexa-His tag. Black line in all panels = pool of mouse sera before immunization (pre-bleed)

Immunization of mice with either D1NS1-HEK or D4NS1-HEK cells resulted in the development of humoral immune responses specific for the NS1 proteins (Fig. 3). Sera of mice immunized with D1NS1-HEK contained higher titers of antibodies against recD1NS1 (Fig. 3a) than against recD4NS1 (Fig. 3c). In contrast, similar antibody titers to both recD1NS1 and recD4NS1 were detected in mice that were immunized with D4NS1-HEK cells (Fig. 3b and d). Individual mouse antisera of the D1NS1-HEK and D4NS1-HEK mouse groups contained similar levels of antibodies to the NS1 target protein. All mouse antisera showed only very low anti-hexa-His tag antibody levels, as determined by ELISA of the sera on an unrelated hexa-His tagged protein (MUL_3720) (Fig. 3e and f).

### Generation and characterization of anti-DENV NS1 mAbs

**Fig. 4:**
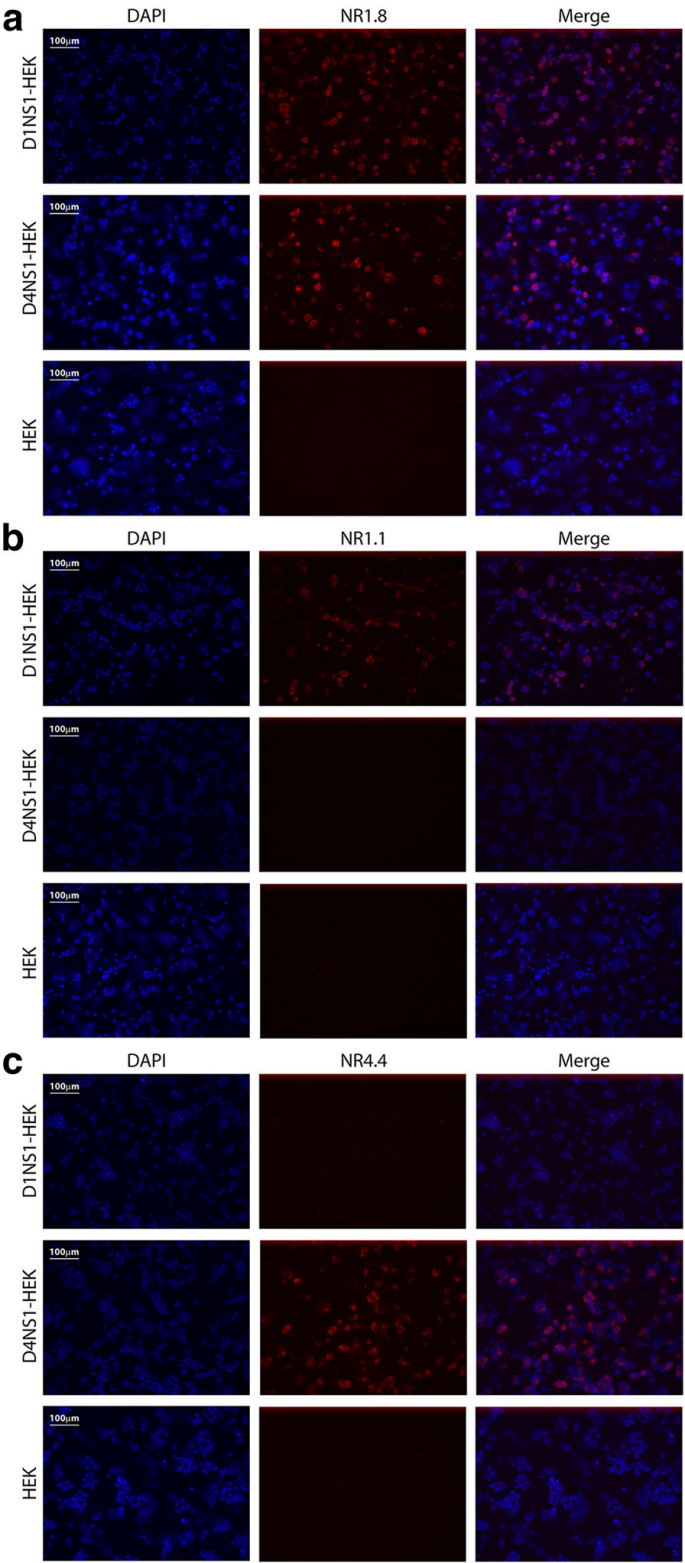
IFA of selected generated anti-NS1 mAbs on HEK cells. IFA of methanol fixed D1NS1-HEK, D4NS1-HEK and untransfected HEK cells after staining with selected generated anti-NS1 mAbs and Alexa568-labelled anti-mouse IgG antibodies. **a**: D1NS1 and D4NS1 serotype cross-reactive mAb NR1.8. **b**: D1NS1 serotype-specific mAb NR1.1. **c**: D4NS1 serotype-specific mAb NR4.4. Nuclei were stained with DAPI

Fusion of spleen cells obtained from mice immunized with D1NS1-HEK or D4NS1-HEK cells with PAI myeloma cells enabled the generation of anti-NS1 antibody-producing hybridoma cell lines. In order to identify cell lines that produce IgG specific for NS1, hybridoma supernatants were screened by ELISA on recD1NS1 or recD4NS1, respectively. Supernatants from ELISA-positive wells were subsequently tested by IFA on D1NS1-HEK and D4NS1-HEK cells. IFA on HEK cells transfected with an unrelated hexa-His tagged protein served as control to identify those hybridoma cell lines that generated NS1-specific antibodies. Screening of D1NS1-HEK and D4NS1-HEK mouse spleen cell fusions yielded eight and four anti-DENV NS1 IgG-producing hybridoma cell lines, respectively. These cell lines were cloned by limiting dilution to obtain hybridoma clones producing the mAbs NR1.1 - NR1.8 and NR4.1 - NR4.4, respectively. Five mAbs were of the IgG1(κ), six of the IgG2a(κ) and one of the IgG2b(κ) IgG subclass (Table 1). IFA with these mAbs on the D1NS1 and D4NS1 transfectants revealed that five specifically recognized D1NS1-HEK but not D4NS1-HEK cells, three only reacted with D4NS1-HEK cells and the remaining four were cross-reactive with both transfectants (Table 1, Fig. 4).

**Table 1:** Characteristics and reactivities of the generated anti-NS1 mAbs

		IFA		ELISA				Western blot analysis			
		Transfected HEK cells		Transfected HEK cell lysates				DENV-infected Vero E6 cell lysates			
mAb	Isotype	D1	D4	D1	D2	D3	D4	D1	D2	D3	D4
NR1.1	IgG1, κ	**+**	**-**	**+**	**-**	**-**	**-**	**+**	**-**	**-**	**-**
NR1.2	IgG1, κ	**+**	**-**	**+**	**-**	**-**	**-**	**+**	**-**	**+**	**-**
NR1.3	IgG1, κ	**+**	**-**	**+**	**-**	**-**	**-**	**+**	**-**	**-**	**-**
NR1.4	IgG2a, κ	**+**	**+**	**+**	**+**	**+**	**-**	**+**	**+**	**+**	**-**
NR1.5	IgG2a, κ	**+**	**+**	**+**	**-**	**-**	**+**	**+**	**-**	**-**	**-**
NR1.6	IgG1, κ	**+**	**-**	**+**	**-**	**-**	**-**	**+**	**-**	**-**	**-**
NR1.7	IgG2a, κ	**+**	**-**	**+**	**-**	**-**	**-**	**+**	**-**	**-**	**-**
NR1.8	IgG2b, κ	**+**	**+**	**+**	**-**	**+**	**+**	**+**	**+**	**+**	**+**
NR4.1	IgG2a, κ	**-**	**+**	**-**	**-**	**-**	**+**	**-**	**-**	**-**	**+**
NR4.2	IgG2a, κ	**-**	**+**	**-**	**-**	**-**	**+**	**-**	**-**	**-**	**+**
NR4.3	IgG1, κ	**+**	**+**	**-**	**-**	**-**	**+**	**-**	**-**	**-**	**+**
NR4.4	IgG2a, κ	**-**	**+**	**-**	**-**	**-**	**+**	**-**	**-**	**-**	**+**

The different recognition patterns of the 12 generated anti-NS1 mAbs as determined by i) IFA on the D1NS1 and D4NS1 transfected HEK cells, ii) ELISA on whole protein lysates of D1NS1-HEK - D4NS1-HEK cells and iii) Western blot analysis on whole protein lysates of DENV-infected VeroE6 cells are summarized

**Fig. 5:**
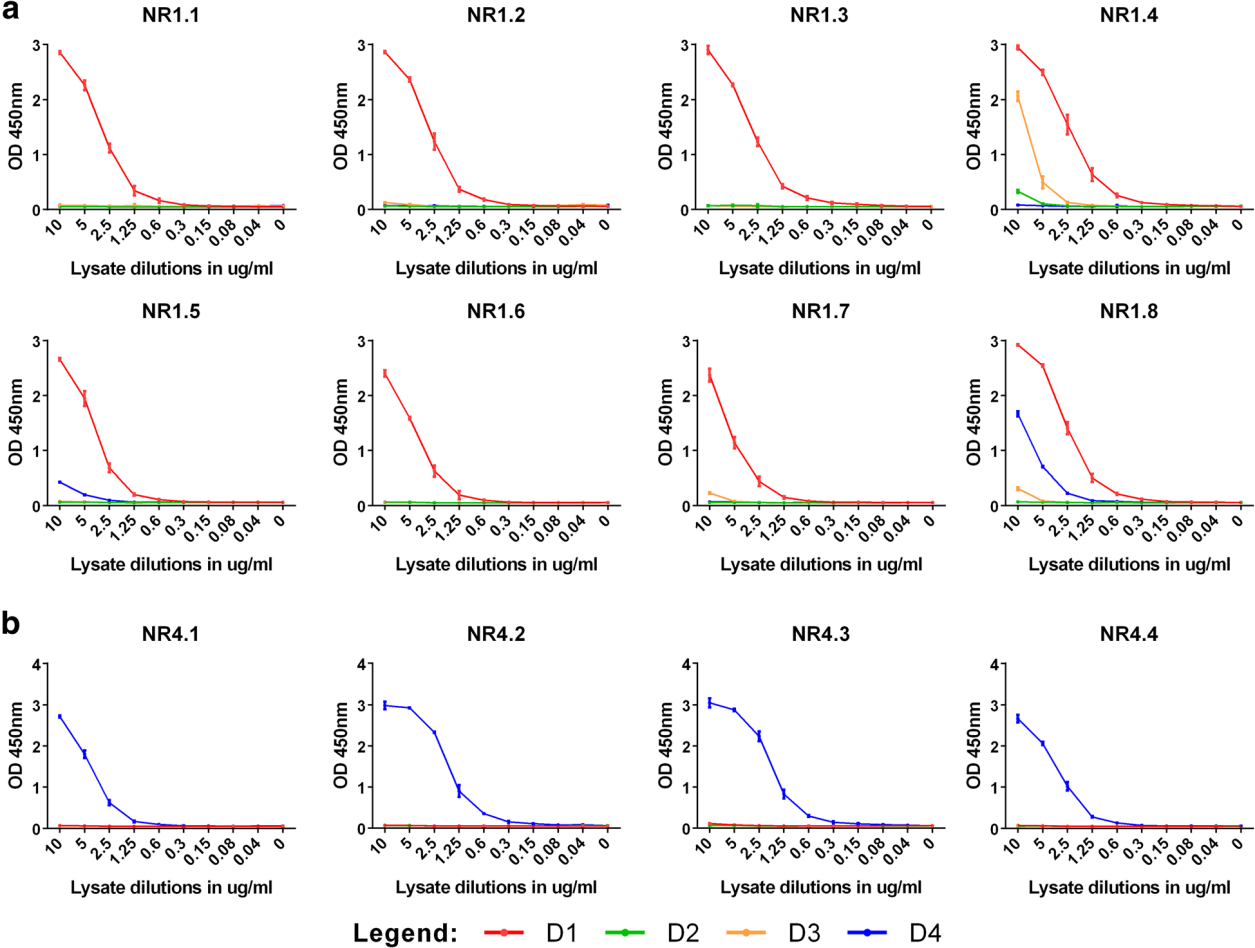
ELISAs of D1NS1-HEK - D4NS1-HEK cell whole protein lysates with the generated mAbs. DENV NS1 serotype-specificity of the 12 generated mAbs was assessed by capturing the NS1 protein in D1NS1 - D4NS1 HEK cell lysates using anti-hexa-His tag mAbs and detecting the protein with the anti-NS1 mAbs NR1.1 - NR1.8 and NR4.1 - NR4.4. **a:** While analyses with mAbs NR1.1 - NR1.3, NR1.6 and NR1.7 exhibited a high degree of D1NS1-specificity in the ELISA, mAbs NR1.4, NR1.5 and NR1.8 showed a certain degree of NS1 cross-reactivity among different DENV serotypes. **b:** MAbs NR4.1 - NR4.4 were highly specific for D4NS1, since they did not recognize D1NS1 - D3NS1 in the HEK cell lysates. M = molecular weight marker in kDa

In order to test serotype-specificity of the generated mAbs on all four DENV serotypes, analyses were extended to HEK cells expressing D2NS1 and D3NS1 (Additional file 2: Figure S2). When serotype specificity was tested by ELISA using anti-hexa-His tag antibodies as common capturing reagent for all four hexa-His tagged NS1 fusion proteins from HEK cell lysates, different recognition patterns were observed for the 12 anti-NS1 mAbs used as biotinylated detecting reagents in ELISA. While five of the eight mAbs raised against D1NS1 (NR1.1, NR1.2, NR1.3, NR1.6 and NR1.7) specifically recognized only D1NS1, three (NR1.4, NR1.5 and NR1.8) also cross-reacted with the NS1 protein of at least one other serotype. In contrast, all four mAbs raised against D4NS1 (NR4.1 – NR4.4) reacted only with D4NS1 in ELISA (Table 1, Fig. 5).

### Detection of virus material derived from DENV-infected VeroE6 cells by the generated mAbs

**Fig. 6:**
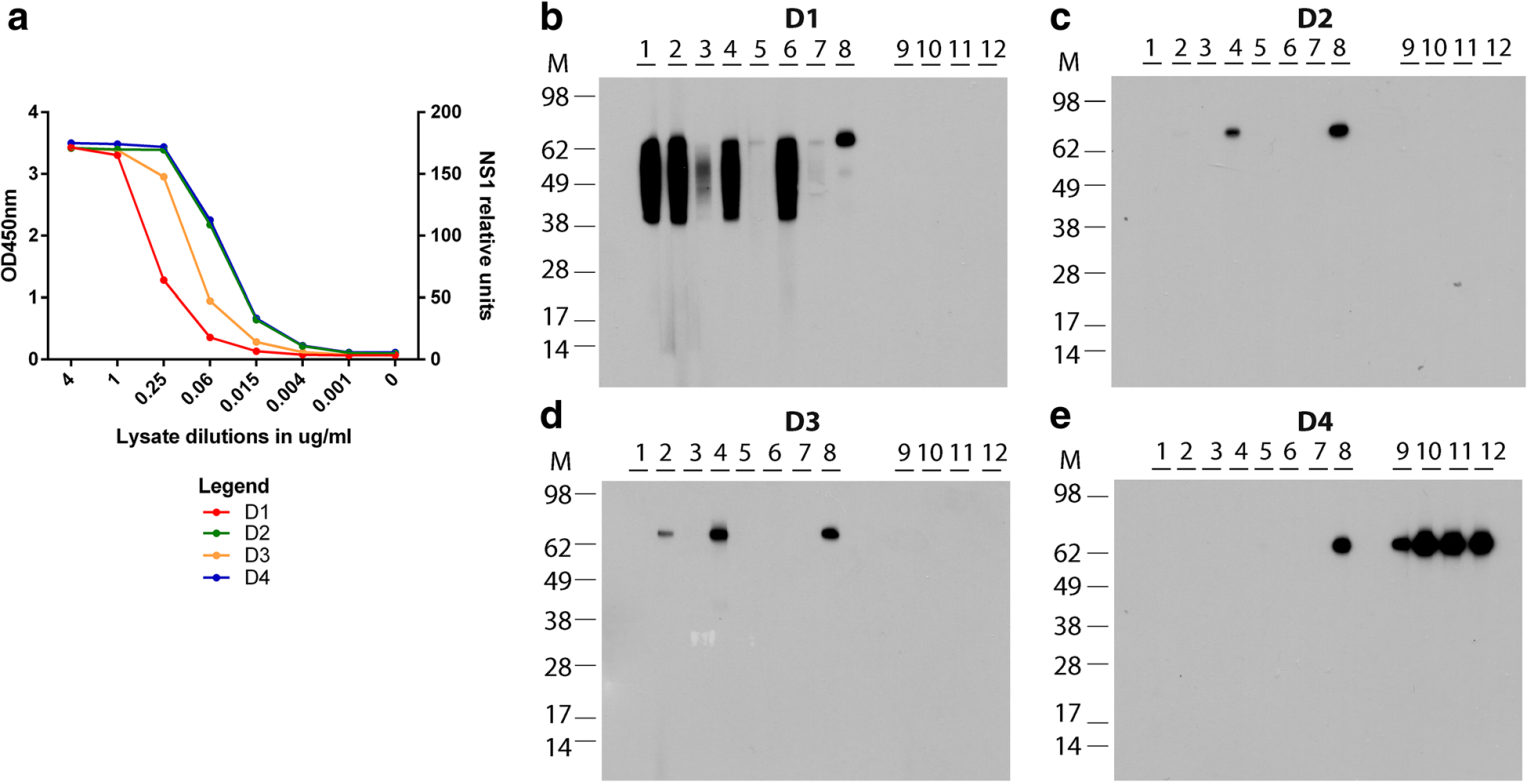
ELISA and Western blot analysis of lysates derived from DENV-infected VeroE6 cell lines. **a**: NS1 relative units were determined in lysates of VeroE6 cells infected with DENV serotypes 1–4 by NS1 capture ELISA (EUROIMMUN, Luzern, Switzerland). NS1 relative units were calculated in accordance with the manufacturer’s instructions and approximate values are shown. Gels for Western blot analysis were loaded with DENV serotype 1–4 infected VeroE6 cell lysates containing 40.000 relative units of NS1, each under non-reducing conditions. The 12 generated mAbs were tested on serotype 1 (**b**), serotype 2 (**c**), serotype 3 (**d**) and serotype 4 (**e**) virus-infected cell lysates

Cross-reactivity of the generated mAbs with virus-infected cells was analyzed by Western blot analysis with lysates of VeroE6 cells infected with the four different DENV serotypes (Fig. 6). Results were nearly identical to the ELISA analysis of all four hexa-His tagged NS1 fusion proteins from HEK cell lysates with three exceptions: i) the Western blot cross-reactive mAb NR1.8 did not react with the D2NS1 protein in the ELISA, ii) mAb NR1.2 did recognize D3NS1 in virus material, but not the NS1 protein present in D3NS1-HEK cell lysates and iii) mAb NR1.5 recognized NS1 in D4NS1-HEK cell lysates, but not the one present in the virus-infected cell lysates. Analysis with lysates of the virus-infected cells showed that mAb NR1.8 is cross-reactive with all four DENV serotypes. Five of the mAbs specifically reacted with D1NS1 and four recognized D4NS1 only. One of the mAbs cross-reacted with D1NS1 - D3NS1, but did not recognize D4NS1 and one reacted with D1NS1 and D3NS1 (Fig. 6, Table 1).

## Discussion

In the present study, we describe an efficient and cost-effective strategy to rapidly generate mouse mAbs against native viral proteins. The methodology used is based on the immunization of mice with HEK cells presenting the native target proteins on their surface. Expression of the proteins on the HEK cell surface is achieved by hijacking the HEK cell’s synthetic protein machinery upon transfection of the cells with a plasmid encoding the target antigen as fusion protein connected to a transmembrane domain [22]. The use of HEK cells as a tool for the expression of native proteins has several advantages. For one, HEK cells are easy to grow in culture, can efficiently be transfected with commercially available transfection reagents, and are also capable of expressing large amounts of recombinant proteins [25]. Moreover, mammalian cell cultures - as opposed to prokaryotic or yeast expression systems – represent an optimal tool for the production of viral proteins (particularly glycoproteins [26]), since protein folding and post-translational modifications closely approximate those occurring in the infected mammalian hosts [27]. The immunization of mice with antigens hooked to the surface of HEK cells has the further advantages that neither elaborate production/processing of recombinant antigen nor addition of adjuvant - that both may negatively affect protein folding - are required.

Here we used this novel approach to rapidly generate panels of mAbs against the DENV NS1 protein. Immunization of mice with D1NS1-HEK and D4NS1-HEK cells, whose viability was preserved by storage at −80 °C in freezing medium, and subsequent production of hybridoma clones yielded D1NS1- and D4NS1 serotype-specific as well as DENV NS1 serotype cross-reactive mAbs. In view of the 30 % variability present among sequences of the four DENV serotypes at the amino acid level (Additional file 1: Figure S1), it was expected that mice immunized with one serotype will develop both serotype-specific and serotype cross-reactive humoral immune responses. In this study a first selection of the desired mAbs was achieved by high-throughput ELISA screening of the hybridoma cell supernatants on small amounts of commercially available DENV D1NS1 - D4NS1 recombinant proteins. However, in order to completely circumvent the use of recombinant proteins, lower throughput screening can also be performed by IFA on HEK cells transfected with the target protein or by Western blot analysis on lysates of transfected HEK cells. These assays should be performed in parallel with tests using material from HEK cells transfected with an unrelated hexa-His tagged fusion protein to exclude hybridomas producing HEK cell and hexa-His tag –specific antibodies from further analysis. Seven of the identified mAbs (the D1NS1-specific NR1.1, NR1.3, NR1.6 ﻿and NR1.7 and the D4NS1-specific NR4.1, NR4.2 and NR4.4) were serotype-specific in all applied assay formats, while one mAb (NR1.8) reacted with the NS1 protein of each of the four serotypes in all but one (ELISA on D2NS1) of the assays. The other four mAbs (NR1.2, NR1.4, NR1.5 and NR4.3) reacted with the NS1 protein of at least two, but not all of the four DENV serotypes (Table 1). Discrepancies in recognition patterns might be explained by steric differences (hindrances) in binding of the mAbs in different assay formats. Western blot analysis of DENV-infected cell lysates confirmed that the generated mAbs can be used to detect the endogenous NS1 protein in DENV-infected biological samples. Differences in recognition patterns might also be explained by sub-serotype specificity of the generated mAbs.

The progressive and ubiquitous threat of newly emerging and re-emerging viral diseases necessitates the development of tools for their prevention, detection and control. In the past decades mAbs have been increasingly used for the development of vaccines, diagnostics and therapeutics as well as for research on the epidemiology of pathogens. In this study we were able to prove our approach of rapidly generating panels of mAbs against native viral proteins. The envisaged production of serotype and sub-serotype variant specific anti-NS1 mAbs against all DENV serotypes will provide various prospects for their application. Antigen capture assays for diagnostic purposes may be developed by using a broadly cross-reactive capturing mAb in combination with different serotype-specific detection mAbs. Variant specific mAbs may be used as tools to study the epidemiology of DENV isolates based on their antigenic diversity [12].

## Conclusions

Our cell-based system of immunizing mice with HEK cells presenting native viral proteins followed by hybridoma selection provides a highly efficient tool to rapidly obtain mAbs against native viral proteins that can be used for various downstream applications. The strategy can easily be extended to a broad range of viral proteins.

## Additional files

Additional file 1: Figure S1. Dengue virus NS1 protein sequences. Sequence alignment of the D1NS1 - D4NS1 protein sequences expressed by the transfected HEK cells showing 30 % sequence variability. Identical amino acid positions among the four serotypes are highlighted in grey. (TIF 2689 kb)
Additional file 2: Figure S2. Expression of D2NS1 and D3NS1 by transfected HEK cells. While aqua staining of lysates prepared from HEK-derived cell lines expressing D2NS1 (D2) and D3NS1 (D3) showed no specific band for the NS1 protein as compared to the lysates of untransfected HEK cells (−) (**a**), Western blot analysis using anti-hexa-His tag antibodies confirmed the expression of D2NS1 and D3NS1 by the HEK cells (**b**). Western blotting using anti-tubulin antibodies was performed as a control for the amount of untransfected and transfected HEK cell lysates loaded on the gels (**c**). M = molecular weight marker in kDa. (TIF 3371 kb)
