# Supplementary figures and images for: Generation of monoclonal antibodies against native viral proteins using antigen-expressing mammalian cells for mouse immunization
Source: BMC Biotechnol. 2016 Nov 22;16:83. doi: 10.1186/s12896-016-0314-5 (PMC5120561; doi:10.1186/s12896-016-0314-5)

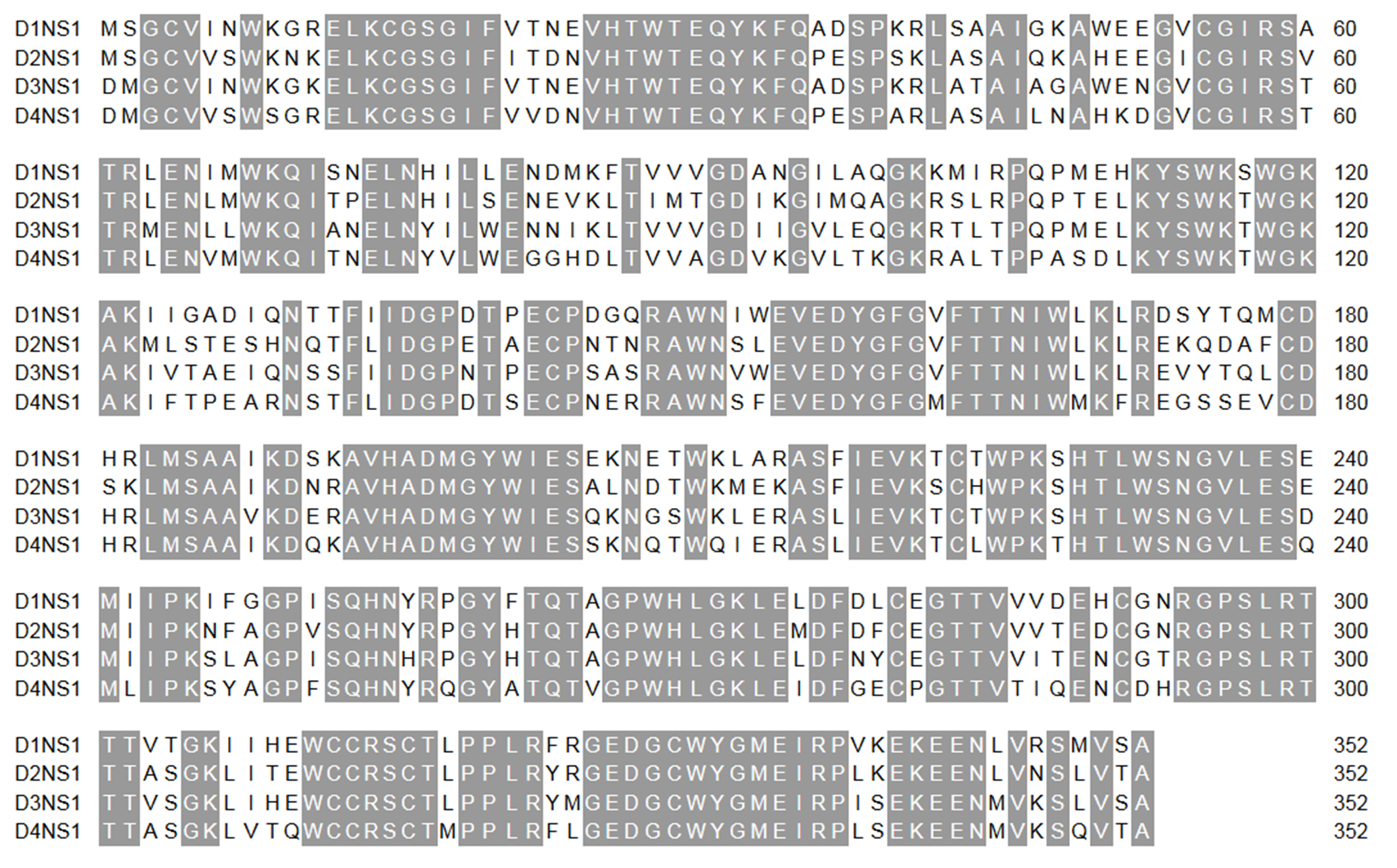

Supplement: Additional file 1: Figure S1. — Dengue virus NS1 protein sequences. Sequence alignment of the D1NS1 - D4NS1 protein sequences expressed by the transfected HEK cells showing 30 % sequence variability. Identical amino acid positions among the four serotypes are highlighted in grey. (TIF 2689 kb) [file 12896_2016_314_MOESM1_ESM.tif]

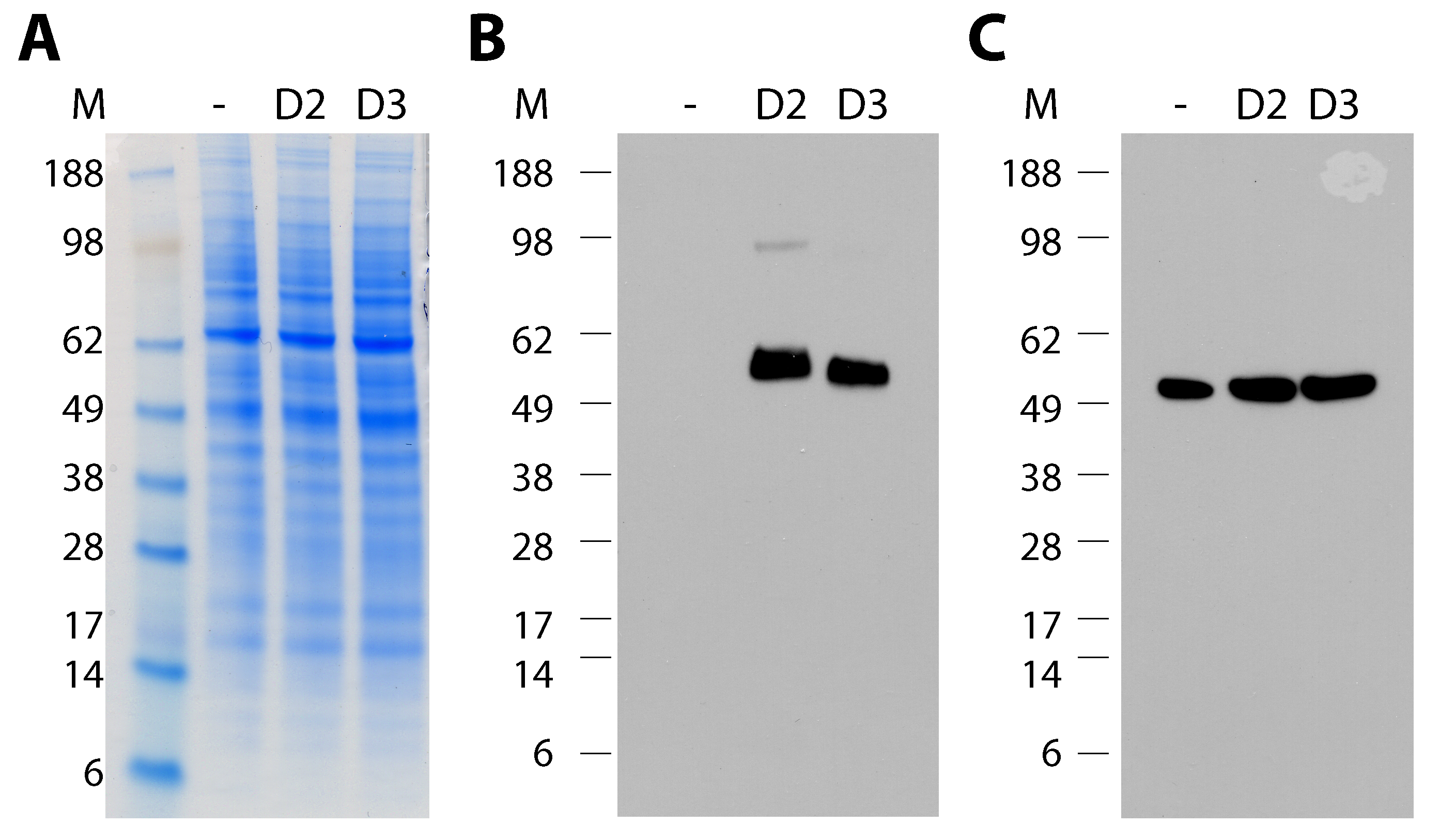

Supplement: Additional file 2: Figure S2. — Expression of D2NS1 and D3NS1 by transfected HEK cells. While aqua staining of lysates prepared from HEK-derived cell lines expressing D2NS1 (D2) and D3NS1 (D3) showed no specific band for the NS1 protein as compared to the lysates of untransfected HEK cells (−) (a), Western blot analysis using anti-hexa-His tag antibodies confirmed the expression of D2NS1 and D3NS1 by the HEK cells (b). Western blotting using anti-tubulin antibodies was performed as a control for the amount of untransfected and transfected HEK cell lysates loaded on the gels (c). M = molecular weight marker in kDa. (TIF 3371 kb) [file 12896_2016_314_MOESM2_ESM.tif]
